# The Role of Streptococcal and Staphylococcal Exotoxins and Proteases in Human Necrotizing Soft Tissue Infections

**DOI:** 10.3390/toxins11060332

**Published:** 2019-06-11

**Authors:** Patience Shumba, Srikanth Mairpady Shambat, Nikolai Siemens

**Affiliations:** 1 Center for Functional Genomics of Microbes, Department of Molecular Genetics and Infection Biology, University of Greifswald, D-17489 Greifswald, Germany; patience.shumba@uni-greifswald.de; 2 Division of Infectious Diseases and Hospital Epidemiology, University Hospital Zurich, University of Zurich, CH-8091 Zurich, Switzerland; Srikanth.MairpadyShambat@usz.ch

**Keywords:** *Streptococcus pyogenes*, group A streptococcus, *Staphylococcus aureus*, skin infections, necrotizing soft tissue infections, pore-forming toxins, superantigens, immunomodulatory proteases, immune responses

## Abstract

Necrotizing soft tissue infections (NSTIs) are critical clinical conditions characterized by extensive necrosis of any layer of the soft tissue and systemic toxicity. Group A streptococci (GAS) and *Staphylococcus aureus* are two major pathogens associated with monomicrobial NSTIs. In the tissue environment, both Gram-positive bacteria secrete a variety of molecules, including pore-forming exotoxins, superantigens, and proteases with cytolytic and immunomodulatory functions. The present review summarizes the current knowledge about streptococcal and staphylococcal toxins in NSTIs with a special focus on their contribution to disease progression, tissue pathology, and immune evasion strategies.

## 1. Introduction

Necrotizing soft tissue infections (NSTIs) are rare and represent a more severe rapidly progressing form of soft tissue infections that account for significant morbidity and mortality [1]. NSTIs can be classified according to the invading organisms (types I–III), and less commonly, the depth of invasion, or anatomic location (trunk, extremity, perineum) [1,2,3,4]. Type I NSTIs, also referred to as synergistic NSTIs, affect around 70%–80% of patients seen in practice [1,3]. They are of a polymicrobial nature, frequently involving a mixture of aerobic and anaerobic bacteria [5] and affect elderly and/or patients with multiple underlying conditions, including diabetes mellitus, obesity, vascular diseases, renal insufficiency, and immunosuppression [6]. Type II NSTIs, causing around 20%–30% of cases, are of a monomicrobial nature mostly due to Gram-positive organisms. Among these, *Streptococcus pyogenes* (group A streptococcus [GAS]) is the most common pathogen [7,8,9,10]. Although *S. aureus* has not been described as a monomicrobial cause of NSTIs in clinical settings until 2005, the number of methicillin-resistant *S. aureus* (MRSA) NSTIs is constantly increasing leading to the second major species responsible for type II NSTIs [11]. Type II NSTIs affect mostly young individuals without underlying conditions with a recent history of trauma to an extremity or intravenous drug abuse [4]. Type III infections are confined to warm coastal areas and are caused mainly by Gram-negative *Vibrio* species [1,12]. This review article focuses solely on type II NSTIs caused by GAS and *S. aureus* and the role of respective exotoxins and secreted proteases contributing to the severity of infection.

## 2. Pathophysiology of Type II NSTIs

**Figure 1:**
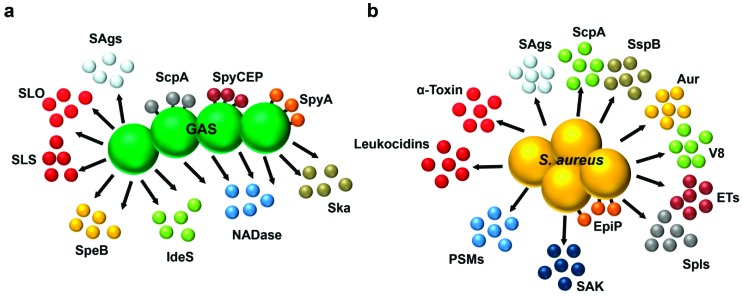
Streptococcal and staphylococcal secreted virulence factors with pore-forming and/or immunomodulatory properties. (**a**) Group A streptococcal (GAS) secreted factors: Streptolysins S and O (SLS, SLO), streptococcal pyrogenic exotoxin B (SpeB), superantigens (SAgs), C5a peptidase (ScpA), Immunoglobulin degrading enzyme of streptococci (IdeS), SpyCEP, SpyA, Streptokinase (Ska), and NADase. (**b**) Staphylococcal secreted factors: Leukocidins, α-toxin, phenol-soluble modulins (PSMs), superantigens (SAgs), staphopain A (ScpA), Staphopain B (SspB), Aureolysin (Aur), V8 protease, exfoliative toxins (ETs), epidermin leader processing protease (EpiP), serine protease-like proteins (Spls), and staphylokinase (SAK).

GAS and *S. aureus* are Gram-positive cocci, which share many features, including clinical aspects and pathogenic mechanisms. Both secrete virulence factors with pore-forming and/or immunomodulatory properties (Figure 1). However, they also have unique features. *S. aureus* is a major cause of community- and hospital-acquired infections ranging from mild superficial skin and throat infections to invasive infections such as toxic shock syndrome (TSS) and NSTIs [13]. A great public health concern is the increasing prevalence of MRSA, specifically the rise in community-acquired (CA) *S. aureus* [13,14,15]. Specifically CA-MRSA clones are associated with highly aggressive infections, including NSTIs, in otherwise healthy individuals [11]. GAS with an estimate of 500,000 deaths annually is rated as number nine on the list of global killer pathogens [16]. GAS can cause a variety of diseases in immunocompetent individuals similar to those listed for *S. aureus* [16].

Type II NSTIs can present with or without a defined portal of entry [4]. In ca. 50% of cases the Gram-positive cocci can gain entry to the deeper tissue (i) after breaches of the skin due to drug injections, incisions or childbirth, (ii) through superficial lesions (e.g., lacerations or insect bites), or (iii) after a penetrating trauma [1]. The proliferation of the bacteria leads to the release of exotoxins, which will cause tissue damage and impair the initial and very crucial inflammatory response. Within the next 24–72 h toxin induced local coagulation disturbances and damage of the endothelium lead to fluid leakage, tissue swelling, and erythema. These changes become widespread leading to the development of bullae, ecchymoses, and further bacterial spread to the deeper layers of the tissue. Further exotoxin production by bacteria leads to occlusion of major vessels with subsequent necrosis of all tissue layers including muscles [4,17]. In the other 50% of cases, NSTIs initiate without a portal of entry, often at sites of non-penetrating trauma (e.g., blunt trauma and bruises) [18]. Tissue injury initiates an influx of leukocytes, activation of myogenic progenitor cells, and trafficking of the microorganisms, by a yet unknown mechanism of initiation, to the affected site [4]. Again, bacteria start to proliferate and produce exotoxins, which leads to the occlusion of arteries. Subsequently, these events result in necrosis of the deeper tissue that spreads to upper tissue layers. In contrast to NSTIs with a defined portal of entry, the bullae and ecchymoses develop later [4].

## 3. Superantigens and Toxic Shock Syndrome

Invasive GAS infections are often complicated by streptococcal toxic shock syndrome (STSS) [19]. According to Sepsis-3 consensus, sepsis is a life-threatening organ dysfunction caused by a dysregulated host response to infection. Toxic shock is a subset of sepsis in which particularly profound circulatory, cellular, and metabolic abnormalities are associated with a greater risk of mortality than with sepsis alone [20]. Approximately 50% of GAS NSTI cases are associated with STSS [21,22], which significantly increases the mortality of GAS NSTIs [21,23]. Although less common, staphylococcal TSS was also reported in cases of skin and soft tissue infections [24]. Staphylococcal TSS is divided in two categories, menstrual and non-menstrual [25]. Menstrual TSS occurs within two days of a woman´s menstrual period and is usually associated with tampon use. Approximately half of the reported cases are of a non-menstrual nature and are reported in a variety of cases, including surgical wound infections, burns, and cutaneous and subcutaneous lesions. The fatality rate of these infections remains around 5% [26].

Toxic shock presents classically in three phases. The first phase is characterized by fever, myalgia, headache, and chills. Nausea, vomiting, and diarrhea may also be present. The second phase expands to systemic manifestations, such as tachycardia, tachypnea, and high fever. In STSS, pain is present in the affected limb, abdomen or thorax. The third phase is characterized by circulatory shock accompanied by multi-organ failure [27]. Both, STSS and staphylococcal TSS are superantigen-driven diseases. However, STSS is a result of an invasive infection (e.g., NSTI), while staphylococcal TSS is secondary to a localized infection (e.g., infections of postsurgical or postpartum injuries, burns, soft tissue injuries, and focal infections) [28]. Superantigens (SAgs) are recognized as key exotoxins mediating the systemic excessive inflammatory response of the host [29]. To date, 26 staphylococcal and 11 streptococcal SAgs were identified [30,31]. *S. aureus* SAgs include the toxic shock syndrome toxin 1 (TSST-1), staphylococcal enterotoxins (SEs) A-E and G-I, and SE-like (SEl) SAgs J-Z [31,32]. The SEs are defined by their emetic activity, while SEls lack this activity or have not been tested yet [32]. TSST-1 was among the first SAgs to be associated with staphylococcal TSS [33]. Streptococcal SAgs include streptococcal pyrogenic exotoxins (Spe) A, C, G-M, streptococcal superantigen (SSA), and streptococcal mitogenic exotoxin Z (SmeZ) [30].

**Figure 2:**
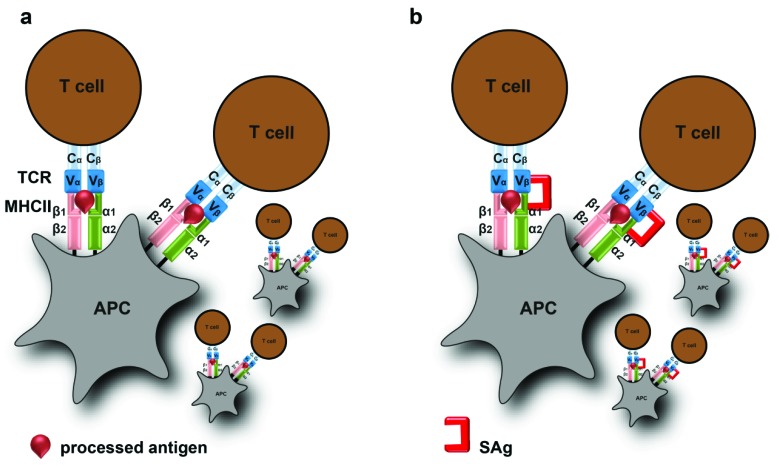
Superantigen (SAg) driven T cell activation. (**a**) Antigen presenting cell (APC) presents a processed antigen peptide on the MHC class II molecule to a T cell via T cell receptor. A process called conventional antigen presentation. (**b**) SAgs bind without cellular processing to MHC class II molecule and variable beta (Vβ) chain on the T cell receptor. This results in uncontrolled T cell activation.

For many years, SAgs were known as pyrogenic toxins based on their common pyrogenic activity [34]. Marrack and Kappler suggested the term superantigen, to emphasize the stimulatory capacity of these exotoxins on T cells [35]. SAgs bind without prior cellular processing to α- and/or β-chains of the major histocompatibility complex (MHC) class II molecules on antigen-presenting cells (APCs) and to the variable β-chains on the T-cell receptor (TCR; Figure 2) [36]. In addition, SAgs can also bind a co-stimulatory molecule CD28 and its ligand CD86 (B7-2) [37,38]. Once the fine MHC-peptide specificity of T cells is bypassed, these interactions result in a massive cytokine storm, including tumor necrosis factor (TNF), interferon (IFN)-γ, interleukin (IL)-1, IL-2, IL-6, CXCL8, CCL2, and CCL3 [39].

The majority of the SAg studies are confined to systemic effects and only a limited number of studies investigated SAg-driven events at the deep tissue site [19,40]. A recent study showed that staphylococcal SAgs TSST-1, SEB, and SEC facilitate the attraction of the adaptive immune system to the local environment through their binding to CD40 on human vaginal epithelial cells [41]. The data suggest that, especially in cases of menstrual TSS, SAgs facilitate infections by the disruption of mucosal barriers and subsequently stimulates chemokine production [41]. Thus, the data indicate that SAg-driven activation of T cells may induce the abundance of homing receptors and thereby promote the migration of activated T cells to the skin or mucosal surfaces. Subsequently, these events lead to exacerbated inflammation of the infected tissue.

## 4. Pore-Forming Toxins

Pore-forming toxins are a class of bacterial virulence factors that disrupt eukaryotic membrane barriers, cause cell lysis, and have immuno-modulatory functions. In this section, we discuss major GAS and staphylococcal pore-forming toxins and their potential implications in NSTIs.

### 4.1. GAS Pore-Forming Toxins

**Figure 3:**
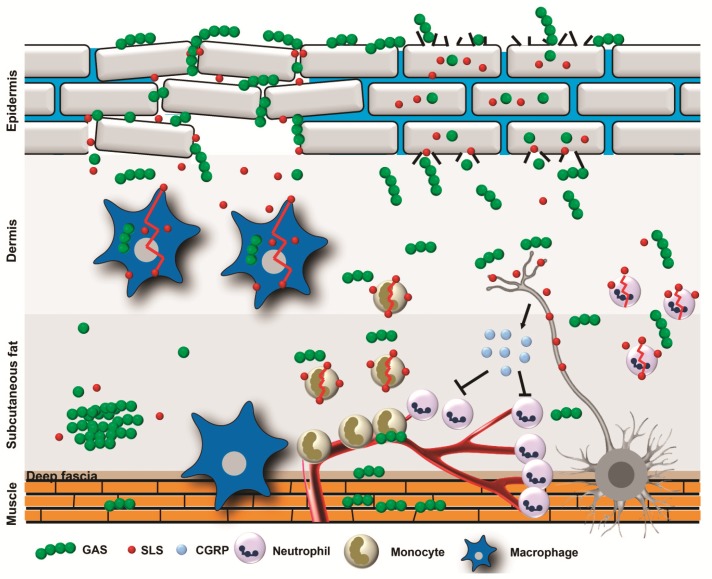
Streptolysin S (SLS) mediated tissue pathology. Group A streptococci (GAS) translocate through the epithelium via cleavage of the junction proteins or direct damage. Once deeper layers are reached SLS stimulates neurons to release calcitonin gene-related protein (CGRP), which inhibits the recruitment of neutrophils. In addition, direct damage of neutrophils, monocytes, and macrophages impairs phagocytic clearance of the bacteria and contributes further to tissue damage. Failed clearance of the pathogen results bacterial dissemination and biofilm formation.

Nearly all clinical GAS isolates secrete a potent hemolysin streptolysin S (SLS) [42]. SLS is a small (2.7 kDa) peptide with the ability to lyse red blood cells, which is typically visualized as a zone of clearance around GAS colonies on blood agar plates, a process called β-hemolysis [43]. SLS is encoded by the SLS-associated gene (*sag*) locus consisting of nine genes (*sagABCDEFGHI*) [44]. The *sagA* gene encodes the premature form of SLS, while the others are required for post-translational modification, processing, and export of the mature SLS [44,45]. SLS is cytolytic only when associated with the bacterial cell surface or carrier molecules [46] and it targets primarily red blood cells, platelets, subcellular organelles, and leukocytes [47,48,49]. It has been suggested that SLS accumulates in cell membranes of eukaryotic cells leading to pore formation and irreversible osmotic lysis of the cells [50], but the exact mechanism is not yet fully understood. A recent study by Higashi and colleagues showed that red blood cell hemolysis by GAS is caused by interactions of SLS with the major erythrocyte anion exchange protein band 3 leading to an osmotic change characterized by a rapid influx of Cl^−^ ions [43]. This finding led the authors to hypothesize that SLS might disrupt similar anion channels in other cell types such as leukocytes, keratinocytes, and endothelial cells [43]. In vivo studies have demonstrated that SLS is a crucial virulence factor in GAS NSTIs [45]. SLS-negative mutants were less virulent in a mouse skin infection model as compared to parental wildtype strains [51], suggesting that SLS expression is detrimental for the pathogenesis of destructive infections. SLS facilitates the translocation of GAS across the epithelial barrier through direct cleavage of junctional proteins occludin and E-cadherin (Figure 3) [52]. In addition, direct damage to keratinocytes is guaranteed via the induction of pyroptosis [53]. An out-of-proportion pain is a critical feature of NSTIs at early stage of infection [1]. A recent study has discovered that SLS activates sensory neurons to produce pain [54]. This leads to a release of neuropeptides that suppress the recruitment of neutrophils to the site of infection and allows the bacteria to spread [54]. Once deeper layers are reached, the direct cytotoxicity of SLS towards a variety of cells residing within the skin tissue and feeding vessels provokes neutrophil influx and further contributes to tissue damage by synergizing with neutrophil-derived factors [55]. Moreover, SLS actively destroys neutrophils which are recruited to the site of infection [56]. This contributes to a negative outcome in patients in two ways; (i) reduced numbers or lack of neutrophils in the infected tissue is an unfavorable prognostic sign in GAS NSTIs [57] and (ii) neutrophil derived effector molecules contribute to hyper-inflammation and tissue damage [19,58,59]. It has also been suggested that SLS impairs the phagocytic clearance of bacteria and further synergizes with other streptococcal virulence factors, such as Streptolysin O (SLO) and the M-protein to augment tissue injury [60]. Failed clearance of the pathogen in deeper tissue layers allows the bacteria to spread and become systemic. In addition, the bacteria can form biofilm, a recently discovered finding in patients suffering from GAS NSTIs [61]. In line with this discovery, Vajjala and colleagues have shown that both streptolysins (SLS and SLO) are required for inducing endoplasmic reticulum stress in the host which, in turn, promotes GAS invasiveness into deeper tissue and biofilm formation [62].

SLO is a 57 kDa oxygen-sensitive, cholesterol-dependent cytolysin, which is encoded by the highly conserved *slo* gene [63]. SLO targets several eukaryotic cells, including macrophages, neutrophils, epithelial cells, and endothelial cells [63]. It disrupts cytoplasmic membrane integrity through pore formation, thereby inducing cell death through pyroptosis [64], apoptosis [65], and necrosis [66]. Despite its cytolytic activity, SLO is able to suppress crucial neutrophil functions at early stages of infection, including migration, oxidative burst, degranulation, release of other pro-inflammatory mediators, and formation of neutrophil extracellular traps (NET) [67]. In addition, SLO contributes to impaired phagocytic clearance of GAS, thereby enhancing bacterial virulence in murine infection models [68]. Zhu and colleagues have shown that a GAS mutant lacking *slo* gene was significantly attenuated in a murine soft tissue infection model [69]. SLO expression is regulated by the two-component system CovR/S, which is known to regulate the expression of up to 15% of the GAS genome [70]. Tissue passage of GAS selects for *cov*R/S mutations resulting in an upregulated SLO expression and further systemic dissemination of the bacteria [71,72]. In addition, Sumby and colleagues have shown that a frameshift mutation in the *covS* gene results in an up-regulation of *slo* transcripts [73]. Furthermore, strains with a non-functional CovR/S TCS were characterized by a higher secretion of SLO, suggesting that CovR/S acts as a repressor of several virulence relevant genes including *slo* [73]. In line with this, it was demonstrated that bacterial isolates derived from invasive human infections show higher SLO activity as compared to isolates from non-invasive infections [74]. Also, antimicrobial peptide LL-37, which is highly abundant in necrotic tissue [75], contributes through the CovR/S system to upregulation of SLO expression and promotes resistance of GAS to killing by human epithelial cells, neutrophils, and macrophages [76]. Moreover, LL-37 promotes vesicle formation by GAS, which contain SLO among other virulence factors, further contributing to GAS pathogenesis [77].

### 4.2. Staphylococcal Pore-Forming Toxins

Alpha toxin (also referred as Hla or Hemolysin-α) is a 33.3 kDa water soluble monomer and is secreted by approximately 95% of *S. aureus* isolates [78]. NSTI-associated CA-MRSA strains tend to express higher levels of this protein as compared to hospital-acquired (HA) MRSA strains [79,80]. Alpha toxin lyses human platelets, endothelial cells, epithelial cells, keratinocytes and leukocytes in two different ways [81]. First, high amounts of the secreted monomeric components integrate via the binding of phosphatidylcholine or sphingomyelin and cholesterol into the membrane of target cells [82,83]. The resultant heptamer structure subsequently leads to the pore formation and lysis of the cells [84]. Second, at lower concentrations, alpha toxin binds A Disintegrin and Matalloprotease 10 (ADAM10) [85] leading to the induction of catalytic activity of the receptor. ADAM10 is a eukaryotic cell surface protease, which is expressed by keratinocytes, endothelial cells, and platelets [86,87,88,89] and whose substrates are members of the notch, ephrin, and cadherin families [87,90,91,92]. The loss of the adherence junctions of the epithelium, e.g., due to the cleavage of E-cadherin, disrupts the epithelial barrier function [93]. Alpha toxin induced pore formation and the resulting Ca^2+^ influx further enhance ADAM10 activity [92,94].

Unlike α-toxin, leukocidins consist of two components and are hetero-oligomeric. Woodin demonstrated for the first time the bi-component composition through the fractionation of the Panton-Valentine Leukocidin (PVL) [95]. Using ion-exchange chromatography, it was shown that PVL consists of a subunit F and S representing fast and slow fractions, respectively [95]. This study showed that the two subunits must be combined to reach the maximum cytolytic activity [95]. The assembled leukocidins are octamers consisting of four F and four *S subunits* [96]. Except of LukAB, which either dimerizes after secretion or is released as a dimer [97,98], the S subunits bind to specific host cell receptors and induce conformational changes to allow dimerization with F subunits, followed by oligomerization of the dimers to form a pre-pore [99]. To date, seven bi-component leukocidins, namely PVL, LukAB, LukED, HlgAB, HlgBC, LukMF´, and LukPQ are described in *S. aureus* (Table 1).

In this review, we limit our discussion to only five, as LukMF´ and LukPQ are circulating in *S. aureus* stains infecting non-human hosts [100,101]. Leukocidins kill human cells and/or modulate the host cell signaling. At higher concentration, the formation of pores ultimately results in cell death. PVL, HlgAB, HlgCB, and LukAB activate the NOD-, LRR- and pyrin domain containing 3 (NLRP3) inflammasome in monocytes and macrophages [102,103,104,105]. Following NLRP3 activation, caspase 1 triggers a pro-inflammatory response and induces pyroptosis [102,103,104,105,106]. At lower toxin concentrations, leukocidins can alter the activation of neutrophils [107,108], trigger the formation of NETs [109], and alter the intracellular signaling in macrophages and neutrophils [102,104,106].

PVL, encoded by the genes *lukF-PV* and *lukS-PV* on bacteriophages [110], was predominantly found (77–100%) in CA-MRSA strains [111,112], which were isolated from skin and soft tissue infections [113,114]. In contrast, less than 3% of colonizing *S. aureus* strains have the PVL genes [115]. It has been difficult to investigate the role of PVL in human infectious diseases. Due to receptor specificity, murine models have been proven to be unreliable to study PVL functions [116]. In contrast, rabbit models have demonstrated to be a useful tool to study diseases, such as necrotizing pneumonia [116]. However, rabbit studies confined to the role of PVL in skin infections contradict each other. While Lipinska and colleagues showed that PVL contributes to tissue pathology in the early stages of infection [117], others could not detect a role of PVL in NSTIs [118]. In contrast to animal models, using a panel of monoclonal antibodies against transmembrane proteins expressed by human neutrophils and macrophages, Spaan and colleagues showed that the human C5a receptors 1 and 2 (C5aR1 and C5aR2) are able to bind the S subunit of PVL and facilitate pore formation [108]. In addition, genome wide CRISPR-Cas9 screen of U937 cells identified human CD45 as a receptor for the F subunit of PVL [119]. CD45 is expressed on all nucleated hematopoietic cells, including T cells, Β cells, and cells of the myeloid lineage [120].

LukAB is a recently discovered leukocidin. Apart from its release into surrounding tissue, it is also present on bacterial surface [98]. The majority of *S. aureus* strains harbor the genes *lukAB* [121], but three out of ten strains fail to express and secrete the protein [122]. The role of LukAB in infections remains elusive. Ex vivo studies showed that LukAB kills human neutrophils by direct interaction with the α-subunit of the αM/β2 integrin (CD11b) [123]. In addition, LukAB can synergize with PVL resulting in cytolytic activity towards monocytes, dendritic cells, and neutrophils [98,104].

LukED is another recently discovered leukocidin. Epidemiological studies showed that about 99% of CA-MRSA strains contain the *lukED* locus, whereas MSSA strains were less likely to contain the genes (44%–77%) [115]. CCR5 was identified as a first LukED receptor by screening the susceptibility of different human cell types, including T cells, macrophages, and dendritic cells [124]. Further analysis identified chemokine receptors CXCR1 and CXCR2 as LukED receptors on neutrophils and monocytes, which were not expressing CCR5 [125]. Together with HlgAB, LukED belongs to the most potent hemolytic leukocidins against human erythrocytes [126]. Both leukocidins target Duffy antigen receptor for chemokines (DARC) to lyse erythrocytes, which, in turn, contributes to *S. aureus* growth due to iron release [126].

γ-Hemolysins (HlgAB and HlgCB) share the same F subunit HlgB, but differ in their S subunit. Both are encoded within the same locus by three genes *hlgABC* [127]. Up to 99% of *S. aureus* strains associated with human colonization express both hemolysins [128]. HlgAB exhibits cytolytic activity towards human red blood cells and leukocytes [129,130], whereas HlgCB is primarily leukotoxic and exhibits only limited cytolytic activity towards red blood cells [131]. As mentioned above, red blood cell lysis is assured through the HlgAB and DARC interaction [126]. In addition, CXCR1, CXCR2, CXCR4, and CCR2 were identified as HlgAB receptors on human neutrophils and macrophages [131]. In contrast, HlgCB interacts with human neutrophils and monocytes via complement receptors C5aR1 and C5aR2 [131].

Phenol-soluble modulins (PSMs) are another class of staphylococcal pore-forming toxins which were discovered first in *S. epidermidis* in 1999 [132]. Eight years later, PSMs were also identified within *S. aureus* core genome [133]. PSMs are divided in two different subfamilies. PSMα peptides (PSMα1- PSMα4 and δ-hemolysin [Hld]) of short amino acid sequence (20–26) are encoded within the *psm*α operon. PSMβs (PSMβ1 and PSMβ2), which are long peptides (40–44 amino acids) are encoded within the psm*β* operon [134]. δ-Hemolysin is encoded within the coding sequence of RNAIII [135]. PSM peptides have a strong impact on the capacity of *S. aureus* to cause skin infections [118,133]. Especially, CA-MRSA strains tend to express higher amounts of PSMs as compared to HA-MRSA strains [118,133]. One of the major contributions of PSMs to *S. aureus* pathogenesis is the ability to lyse eukaryotic cells. In contrast to α-toxin and bi-component leukocidins, it is most likely a receptor independent process [136]. PSMα peptides have the strongest ability to lyse erythrocytes and leukocytes, Hld has moderate cytolytic activity, and PSMβ peptides are not cytolytic [137]. Several studies have demonstrated that PSMα peptides facilitate killing of osteoblasts [138] and neutrophils after phagocytosis [139,140]. At sublytic concentrations, PSMα4 initiates pro-inflammatory responses, including chemoattraction and activation of neutrophils leading to a release of CXCL8 [133,136] and heparin-binding protein, which further induces vascular leakage [141]. PSMα1, PSMα3, and Hld can also induce mast cell degranulation [142] and stimulate IL-10 production by human dendritic cells, which in turn suppresses secretion of pro-inflammatory cytokines [143]. Consequently, these dendritic cells favor priming of regulatory T cells with suppressor function, thereby impairing the Th1 response [143]. Recently, it was also shown that PSMα triggers cutaneous inflammation [144]. The release of IL-1α and IL-36α by keratinocytes drives IL-17 production by γδ T cells and type 3 innate lymphoid cells (ILC3) leading to neutrophil recruitment to the site of infection [144].

Although several virulence factors are implicated in contributing towards fulminant NSTIs, each exotoxin might play a certain redundant and/or non-redundant role in eliciting tissue damage and inflammation. During NSTIs, several of these secreted virulence factors might be co-expressed and in turn collectively contribute towards fulminant infections. The cell specificity of these several toxins may play a major role in the coordinated action of the toxin-induced tissue damage. For example, α-toxin and PVL can synergize. Alpha toxin induces the direct cytolytic effect towards epithelial cells which will result in CXCL8 release and subsequent neutrophil chemotaxis. The presence of PVL will activate and lyse recruited neutrophils exacerbating the tissue damage [106,145]. This phenomenon has been mainly shown in experiments using lung epithelial cells. A similar mode of action might also be relevant in NSTIs. Similarly, both PVL and LukAB can individually cause neutrophil lysis, but their cytotoxic effect is further enhanced when combined together [98,106]. In addition, SAg-translocation (e.g., TSST-1) is augmented by α-toxin and leukocidins which further enhances inflammation of the epithelium and contributes towards epithelial barrier disruption [146,147]. Although this coordinated effect of toxin synergism has not been empirically tested during NSTIs, focusing only on individual toxins can definitely obscure the co-operative actions during infection. Hence, further studies focusing on toxin synergisms during NSTIs need to be conducted. Potentially, these combined effects on specific cell types can amplify tissue pathology to the benefit of the invading bacteria and may define the disease severity and clinical outcome.

## 5. Proteases and Other Immune-Modulatory Toxins

Proteases are secreted virulence factors which promote establishment of infection through damage of barriers. They inhibit transmigration of immune cells to the site of infection and suppress their function. In this section, we discuss major streptococcal and staphylococcal proteases and their implication in NSTIs.

### 5.1. Streptococcal Proteases and Other Toxins

Despite its name, streptococcal pyrogenic exotoxin B (SpeB), SpeB is neither pyrogenic nor an exotoxin. SpeB is a cysteine protease and one of the first proteases identified in GAS [148]. The *speB* gene is highly conserved in all GAS strains [149]. The gene encodes a zymogen of 40 kDa that is autocatalytically cleaved into a mature 28 kDa protein [150]. SpeB cleaves a broad spectrum of streptococcal and human host proteins. On the bacterial site, SpeB is able (i) to remove proteins from the surface, which includes M-protein, fibronectin-binding proteins, and C5a peptidase [151,152,153] and (ii) to hydrolyze secreted proteins, such as streptokinase, EndoS, SLO, and SAgs [154,155,156,157]. On the host site, SpeB cleaves IgG into Fc and Fab fragments and degrades IgA, IgM, IgD, and IgE [158]. The cleavage of IgG results in impaired opsonophagocytosis and increased survival of GAS in human blood [159]. Further, SpeB cleaves components of the complement activation pathway. Kuo and colleagues demonstrated that C3b is cleaved by SpeB leading to impaired phagocytic killing of bacteria by neutrophils [160]. In support of this, Terrao and colleagues detected only degraded C3b fragments in sera of patients diagnosed with STSS [161]. Moreover, SpeB degrades a wide range of chemokines, including, CXCL1, CXCL2, CXCL3, CXCL4, CXCL5, CXCL6, CXCL7, CXCL10, CXCL11, CXCL12, CXCL13, CXCL14, CXCL16, CCL20, XCL1, and CX3CL1 [162] and cleaves pro-IL-1β into biologically active IL-1β [163]. It was suggested that IL-1β, which activates the NLRP3 inflammasome acts as a sensor of intracellular proteolytic activity of SpeB [164]. Moreover, IL-1β pathway plays a key role in modulating susceptibility of the host to GAS NSTIs [165]. SpeB also interferes with coagulation and anticoagulation pathways by degrading fibrinogen and plasmin, respectively [166,167] and contributes to tissue pathology via the degradation of extracellular matrix proteins and the activation of matrix metalloproteases [168,169].

Although SpeB shows such a broad spectrum of substrates, its role in invasive GAS infections is still controversial. The s*peB* gene can be found in isolates from all types of diseases [170,171]. Some studies show that SpeB is readily detectable in patients´ sera and tissues [61,172]. Others demonstrate that SpeB amounts and activity produced by isolates from non-severe cases are higher as compared to isolates from severe cases [173]. However, low anti-SpeB antibody titers have been associated with severe diseases [174]. This controversy continues also in interpretation of the results generated from mice models. While some authors report that SpeB contributes to disease severity, mortality, bacterial dissemination, and tissue damage [175,176,177,178], others show that *speB*-deficient strains are as virulent as the parental wild type strains [179,180]. Loss of SpeB expression through mutations in *cov*R/S or *ropB* is believed to trigger a hyper-virulent phenotype of bacteria [72]. However, human tissues from NSTIs cases are strongly positive for SpeB [61,75]. As recently shown, most likely it is a mixed population of SpeB-positive and SpeB-negative clones contributing to tissue pathology and disease severity [61].

Immunoglobulin degrading enzyme of *S. pyogenes* (IdeS) is a 35 kDa secreted cysteine protease which hydrolyses four subclasses of human IgG [181]. As a consequence, bacterial bound IgGs that are cleaved by IdeS lack IgG-Fc receptor and complement binding/activation capability. Apart from its implications as an important anti-phagocytic virulence factor [182], the role of IdeS in NSTIs is not yet clear.

GAS express two major subtilisin-like serine proteases with immunomodulatory functions, C5a peptidase (ScpA) and SpyCEP. ScpA contains an LPXTG motif which facilitates anchoring of the protein to the bacterial cell wall [183]. Until recently, the human anaphylatoxin C5a was reported as the only substrate for ScpA [184]. The cleavage of C5a results in impaired neutrophil activation and recruitment to the site of infection [185]. Recently, Lynskey and colleagues identified C3 and C3a as novel substrates for ScpA [186]. Cleavage of C3a leads to impaired human neutrophil activation, phagocytosis, and chemotaxis, while cleavage of C3 generated C3a and C3b fragments with impaired functions [186]. SpyCEP is a 180 kDa, surface-exposed, subtilisin-like serine protease that helps GAS to disseminate in soft tissue [187,188]. SpyCEP is highly expressed in vivo [189] and cleaves CXC chemokines, including CXCL1, CXCL2, CXCL3, CXCL5, CXCL6, and CXCL8 [187,188,190], which results in an impaired chemoattraction of eosinophils, neutrophils, and monocytes to the site of infection [189,191]. Moreover, SpyCEP promotes resistance to phagocytic clearance of bacteria by reducing formation of NETs [189]. Recently it was also shown that functional SpyCEP is detrimental for invasion of human epithelial and endothelial cells and for biofilm formation [192].

NAD-glycohydrolase (NADase) is encoded by the *nga* gene and is co-transcribed with the *slo* gene [193]. NADase cleaves NAD in mammalian cells, thereby promoting cytotoxicity through the depletion of energy sources [194]. Several in vivo and in vitro studies have demonstrated synergistic toxicity by SLO and NADase in GAS infections [69,193]. A recent study suggests that binding of NADase to SLO stabilizes both toxins, thereby increasing host cell toxicity [195].

SpyA is a 25 kDa surface exposed C3-like ADP-ribosyltransferase which catalyzes the transfer of an ADP ribose moiety of NAD^+^ to target proteins [196,197,198]. It is believed that SpyA modifies actin, vimentin, and tropomyosin to disrupt cytoskeletal structures and promote colonization of the host [196]. In addition, SpyA induces pyroptosis in macrophages, resulting in a release of IL-1β, which in turn enhances bacterial clearance [199].

Streptokinase (Ska) is a plasminogen activator protein which non-enzymatically converts plasminogen to proteolytically active plasmin [200]. To date, Ska has been found in all GAS isolates. The molecule is comprised of three domains (α, β, and γ) and three distinct *ska* alleles, type 1, 2a, and 2b have been described [201]. The majority of GAS strains isolated from skin infections are harboring type 2b *ska* allele [201]. Although Ska activates plasminogen, it is not a protease. GAS cover their surface via different surface anchored or surface associated virulence factors with plasminogen, which, in turn, leads to acquisition of streptokinase [202,203]. The Ska-plasminogen interaction leads to exposure of an active site in the complex, which results in a proteolytical conversion of plasminogen to plasmin [204,205]. Due to host-specificity of Ska, GAS are exclusively human pathogens, no differences in virulence between wildtype and *ska*-deficient GAS mutants are seen in murine infection models [206]. In humanized transgenic mice, expressing human plasminogen, the mortality of mice infected with *ska*-mutant is largely abrogated [207]. In line with this, the SpeB-negative M1T1 GAS variant 5448AP expresses higher levels of Ska as compared to the parental strain 5448 and shows higher surface plasminogen acquisition resulting in hyper-virulence in a subcutaneous infection model of humanized transgenic mice [72].

### 5.2. Staphylococcal Proteases and Other Toxins

Staphylococcal cysteine proteases are papain-like proteases that belong to the C47 family of cysteine peptidases. They can directly or indirectly damage the epithelium as well as connective tissue [208]. Two cysteine proteases, staphopain A (ScpA) and staphopain B (SspB), were identified in *S. aureus*. ScpA is a 20 kDa protein, which auto-activates upon release into environment [209]. Its broad spectrum of substrates includes collagen, elastin, fibronectin, fibrinogen, and kininogen [210,211]. In addition, ScpA blocks CXCR2 on neutrophils via cleavage of the N-terminal domain, making neutrophils unresponsive to activation by all CXCR2 ligands [212]. Moreover, this cleavage results in impaired neutrophil migration towards CXCR2 chemokines [212]. SspB is a 20 kDa peptidase, which is structurally related to ScpA [209]. SspB cleaves CD11b on monocytes and neutrophils resulting in an atypical cell death [213]. Moreover, SspB blocks phagocytosis of *S. aureus* by neutrophils and monocytes and represses their chemotactic activity by a yet unknown mechanism [214].

The group of staphylococcal serine proteases encloses three major classes: the SspA (or V8 protease), epidermin leader peptide processing serine protease (EpiP), and exfoliative toxins (ETs). V8 protease is secreted as an inactive precursor and requires aureolysin (Aur) for its maturation [209]. The mature V8 protease degrades the Fc region of immunoglobulins leading to impaired interaction of immune effector cells with the antigen [215]. In skin infections, V8 protease disrupts the structure of the *stratum corneum* but does not cause epidermal hyper-proliferation or inflammatory cell infiltration [216]. The role of EpiP in *S. aureus* pathogenesis is not fully understood. EpiP is a subtilisin-like serine protease that cleaves collagen [217]. Mice vaccinated with EpiP were protected from subcutaneous *S. aureus* infection [217]. As mentioned above, its structural homologue in *S. pyogenes*, SpyCEP, inactivates CXCL8 and impairs the recruitment of neutrophils to the site of infection [187,189]. However, whether EpiP has similar pathogenic mechanisms remains to be investigated. The third class of serine proteases are the epidermolytic ETs. Although not involved in severe skin infections, ETs can cause breakage of the upper layers of the skin [218]. Four ETs, namely ETA, ETB, ETC, and ETD are known so far [218]. However, ETA and ETB are implicated in human skin infections [219], while ETC and ETD are more related to non-human hosts. Both, ETA and ETB cleave desmoglein 1, a glycoprotein responsible for cell-cell adhesion of the keratinocytes in *stratum granulosum* without affecting E-cadherin [220]. In addition to serine proteases, *S. aureus* secretes six serine protease-like proteins (SplA-SplF) [221], which show amino acid homology with SspA and ETs [222]. In contrast to other serine proteases, Spls are mainly implicated in allergic airway reactions such as asthma [223].

Aureolysin (Aur) belongs to the family of zinc-dependent metallopeptidases [224]. In vitro, it was shown that Aur cleaves α1-protease inhibitor, which is responsible for regulation of neutrophil elastase [225]. In line with this, Burlak and colleagues demostrated that Aur is expressed within phagocytic vacuoles of human neutrophils [226]. Moreover, Aur can cleave the antimicrobial peptide LL-37 [227] and complement component C3 to C3b [228]. As a result, *S. aureus* is poorly opsonized leading to attenuated phagocytosis and bacterial killing [228].

Staphylokinase (SAK) is a secreted and cell surface associated virulence factor of staphylococci and is structurally unrelated to streptokinase [229]. Especially clinical *S. aureus* isolates of skin and mucosal origin express high levels of SAK [230]. SAK stimulates the production of human antimicrobial peptides (LL-37 and α-defensins), binds, and inactivates their bactericidal properties [231,232]. However, the main SAK activity affects its ability to convert plasminogen to an active proteolytic enzyme plasmin [230]. First, *S. aureus* binds plasminogen via surface expressed proteins (e.g., FnBPA and FnBPB) and second, SAK activates plasminogen to plasmin, thereby creating a bacteria-bound serine protease activity [233]. These events enable the bacteria to degrade immunoglobulin G (IgG) and C3b, thereby contributing to immune evasion [234].

### 5.3. Two Component Systems and Exotoxin Regulation

During bacterial infections the regulation of exotoxins is mediated by a complex network which incorporates environmental signals towards coordinated responses against host microenvironment. Two component systems (TCS) are one such mechanism adopted by bacteria. An external signal activates the membrane bound histidine kinase. This induces auto-phosphorylation and downstream activation of a response regulator by its phosphorylation. The binding of the regulator to specific DNA sequence results in its gene expression. Most important and well-studied TCS are AgrAC and SaeRS in *S. aureus* and CovR/S in GAS strains. Both are known to regulate the virulence factors that mitigate the host responses during fulminant NSTIs [235,236].

Differential gene regulation of exotoxins by TCS determines the specificity of toxin gene expression at the site of infection. *S. aureus* exotoxins are upregulated in a growth density dependent manner during NSTIs [237,238]. Furthermore, leukocidins are found to be upregulated during NSTIs [239]. These virulence factors are mainly regulated by the intracellular effector responses belonging to *agr* quorum-sensing system including the transcriptional regulators AgrA and RNA III [240,241,242]. The *agr* system governs the expression of secreted virulence factors and exotoxins which enhance acute infection and bacterial dissemination [240,241,242]. Virulence factor production is mainly regulated through two pathways: (i) RNAIII-dependent synthesis of exotoxins and inhibition of cell surface factors and (ii) an RNAIII-independent, AgrA-mediated production of PSMs and metabolic genes. However, mutations in the *agr*-operon rendering dysfunctional Agr system are associated with adaptation of the bacteria to host environment and inducing a more persistent phenotype [243]. Such Agr-defective systems are usually detected in colonizing strains and in strains isolated from patients diagnosed with endocarditis or bacteremia [244]. Similarly, a point mutation in the *agrC* region was associated with cytotoxic versus colonizing properties of *S. aureus* phenotypic variants causing skin and soft tissue infections [245]. These data further support the importance of virulence regulation and its impact on clinical presentation. Enhanced virulence expression mediated by active Agr system is usually detected during severe invasive and acute infections such as NSTIs [246], whereas *agr*-mutants are usually implicated in causing dormant state and chronic infections, such as endocarditis and osteomyelitis [240]. In addition to the Agr system, the *S. aureus* exoprotein expression system (SaeRS) plays also an important role in regulating virulence factor production at the tissue site. SaeRS consists of the histidine kinase SaeS and the response regulator SaeR. SaeR activates transcription of the downstream target genes [247]. The activated SaeRS TCS induces the expression of several virulence factors, including α-toxin, β- and γ-hemolysins, PVL, TSST-1, and exfoliative toxins [248,249]. Human neutrophil peptides 1, 2, and 3 (HNP1-3), which are located in azurophilic granules of neutrophils and calprotectin, a cytoplasmic neutrophil peptide, activate the SaeRS system [250]. Therefore, neutrophil-mediated activation may play a pivotal role in exotoxin regulation and toxin production at the tissue site. It was proposed that SaeRS TCS acts downstream of Agr in virulence regulation and toxin production pathways [251]. However, the exact mechanism of a relationship between Agr and SaeRS systems is still not fully understood. Moreover, recent studies have implicated that the Agr and SaeRS are independent systems of toxin regulation [247,252,253].

Recent evidence indicates that GAS invasiveness is instigated by spontaneous mutations of the CovR/S TCS [61,72,73,254]. CovR/S is a negative transcriptional regulator of around 15% of the GAS genome. It was shown that mice tissue passage of GAS selects for a 7-bp frame-shift mutation in the *covS* gene encoding the sensor kinase component and this in turn promotes GAS invasiveness [61,72,73]. Since that discovery several investigators reported the role of CovR/S system in severe invasive infections. Mutations in that particular region result in the upregulation of several secreted virulence factors, including a bacteriophage-encoded DNase [73], SLO [71], and SpyCEP [73]. In addition, dysfunctionality of CovR/S results in loss of SpeB expression. Whether the loss of SpeB and/or enhanced expression of other secreted virulence factors are beneficial for the bacteria or not, was discussed earlier in this article. However, the role of the host tissue micro-environment and the availability of nutrients, which influence the expression of response regulators during NSTIs is still not fully understood. Future studies focused towards understanding the interplay between the signaling pathways will be essential to better understand the physiological significance of toxin expression in the context of host tissue micro-environment during NSTIs.

## 6. Treatment

The management of NSTI patients includes fluid resuscitation, support of failing organs, rapid surgical debridement of infected tissue, broad spectrum antibiotics, and adjuvant intravenous polyspecific immunoglobulin G (IVIG) and/or hyperbaric oxygen (HBO) therapy [1,2,255,256,257]. Aggressive tissue debridement guarantees elimination of the necrotic tissue and the source of infection and exotoxins. Recent studies suggest that early surgical intervention within 24 h post admission significantly improves the survival of patients [258,259]. Survival further increases if debridement is performed even earlier [260,261].

Nearly all GAS are susceptible to penicillin. However, the high bacterial load in the tissue results in most GAS being in the stationary or in biofilm stage, making cell-wall active antimicrobials not always effective [61,262]. Therefore, treatment with clindamycin, a protein synthesis inhibitor, in combination with penicillin is strongly recommended [257]. However, clinical data based on randomized trials are lacking. Clindamycin inhibits production of SAgs [263] and a recent observational study showed that clindamycin improves survival of patients with STSS [264]. Nonetheless, experimental data suggest that sub-inhibitory concentrations of clindamycin enhance expression and activity of SLO in vitro, but suppress the expression of SpeB [265,266]. In addition, the rise of clindamycin resistant GAS strains [267] raises concerns about the benefits of clindamycin treatment.

When MRSA is suspected, i.v. linezolid or daptomycin may be added in preference of vancomycin, as the latter has no effect on exotoxin production [1,257]. In addition, poor tissue penetration of vancomycin lowers its efficacy in severe NSTIs [268]. Linezolid, an oxazolidinone, inhibits bacterial exotoxin production [269] and several studies concluded that linezolid is an effective alternative to vancomycin for treatment of skin infections caused by MRSA [270,271,272]. In contrast, daptomycin is a cyclic lipopeptide with a distinct mechanism of action. It inserts into the cell membrane of bacteria via phosphatidylglycerol and disrupts membrane integrity by extracting lipids resulting in ion leakage [273]. Overall, inhibitors of toxin production, such as clindamycin, linezolid or rifampicin are commonly recommended for inclusion in antimicrobial treatment of necrotizing infections.

The use of IVIG and HBO as adjunctive therapies is still under debate. Experimental data showed that IVIG neutralizes bacterial exotoxins, including streptococcal and staphylococcal SAgs [274,275,276,277], α-toxin [145], bi-component leukocidins [145], and SLO [278] among others. However, clinical studies contradict each other. One of the first studies in seven patients with severe NSTI caused by GAS suggested a beneficial role of IVIG [279]. A prospective observational study conducted in STSS patients showed reduced mortality in patients receiving IVIG, while a sub-analysis of the NSTI patients did not confirm this observation [264]. In line with this, a recent placebo-controlled clinical trial called INSTINCT, showed no benefit of the IVIG use in NSTIs [255]. The latest systematic review and meta-analysis of the previous single randomized and four nonrandomized studies revealed that administration of IVIG to clindamycin treated patients is associated with a significant reduction in mortality [280]. Same contradiction applies to adjunctive HBO treatment. Two recent studies concluded that HBO treatment is associated with significant reduction in mortality in NSTIs [7,281]. Nevertheless, a systematic literature review of 57 studies revealed that HBO is not useful for the treatment of NSTIs [282]. Currently, a study delineating the effects of HBO on biomarkers in NSTIs is being performed in Denmark [283].

## 7. Conclusions

NSTIs are rapidly progressing, life-threatening necrotic infections of any layer of the soft tissue compartment. The underlying mechanisms of these infections are poorly understood. GAS and *S. aureus* are equipped with an arsenal of virulence factors that contribute to disease pathogenesis. In NSTIs, there is a clear correlation between exotoxin production at the site of infection and tissue pathology and systemic toxicity. Therefore, secreted virulence factors, including SAgs, pore-forming toxins, and immunomodulatory proteases, are attractive targets for therapeutic approaches. However, further understanding of mechanistic actions of the exotoxins in vivo and in vitro is needed.

## Figures and Tables

**Table 1:** *S. aureus* leukocidin receptors and human target cells.

Leukocidin	Other Names	Receptors	Human Cell Targets
PVL	PVL-LukSV	C5aR1 C5aR2	Neutrophils Monocytes Macrophages
LukAB	LukGH	CD11b	Neutrophils Monocytes Macrophages Dendritic cells
LukED		CCR5 CXCR1 CXCR2 DARC	T cells Neutrophils Monocytes Dendritic cells Erythrocytes
HlgAB	γ-hemolysin γ-toxin	CXCR1 CXCR2 CXCR4 CCR2 DARC	Neutrophils Monocytes Macrophages Erythrocytes
HlgCB	Leukocidin γ-hemolysin γ-toxin	C5aR1 C5aR2	Neutrophils Monocytes Macrophages
